# Associations between comorbid anxiety and sleep disturbance in people with bipolar disorder: Findings from actigraphy and subjective sleep measures

**DOI:** 10.1016/j.jad.2022.04.065

**Published:** 2022-07-15

**Authors:** Daniel J. Oakes, Holly A. Pearce, Cerian Roberts, Phillip G. Gehrman, Catrin Lewis, Ian Jones, Katie J.S. Lewis

**Affiliations:** a National Centre for Mental Health, Division of Psychological Medicine and Clinical Neurosciences, School of Medicine, Cardiff University, UK; b Cardiff University School of Medicine, Cardiff, UK; c Perelman School of Medicine, University of Pennsylvania, Philadelphia, USA

**Keywords:** NCMH, National Centre for Mental Health, NHS, National Health Service, BD-I, bipolar disorder (type I), BD-II, bipolar disorder (type II), MINI, Mini International Neuropsychiatric Interview, PD, panic disorder, GAD, generalised anxiety disorder, PSQI, Pittsburgh Sleep Quality Index, AMS, Altman Self-rating Mania Scale, BDI-II, Beck Depression Inventory (Version II), PHQ-9, Patient Health Questionnaire (9 Item), TST, total sleep time, SOL, sleep onset latency, SE, sleep efficiency, WASO, wake after sleep onset, EEG, electroencephalography, Bipolar disorder, Sleep, Anxiety disorders, Comorbidity, Actigraphy

## Abstract

**Background:**

Studies show that comorbid anxiety disorders are common in people with bipolar disorder. However, little is known about whether this anxiety is associated with sleep disturbance. We investigated, in individuals with bipolar disorder, whether comorbid anxiety disorder is associated with sleep disturbance.

**Methods:**

Participants were 101 (64% female) currently euthymic individuals with a history of bipolar disorder. Sleep disturbances were assessed using self-report measures of sleep quality (Pittsburgh Sleep Quality Index, PSQI) and six weeks of sleep monitoring using actigraphy. Bipolar disorder and comorbid anxiety diagnoses were assessed using the Mini International Neuropsychiatric Interview. Multiple regression analyses examined associations between comorbid anxiety and sleep disturbance, whilst controlling for confounding covariates known to impact on sleep.

**Results:**

A comorbid anxiety disorder was associated with increased sleep disturbance as measured using the PSQI global score (*B* = 3.58, 95% CI 1.85–5.32, *P* < 0.001) but was not associated with sleep metrics (total sleep time, sleep onset latency, sleep efficiency, and wake after sleep onset) derived using actigraphy.

**Limitations:**

Objective measures of sleep were limited to actigraphy, therefore we were not able to examine differences in sleep neurophysiology.

**Conclusions:**

Clinicians should be aware that comorbid anxiety may increase the risk of experiencing subjective sleep disturbance in people with bipolar disorder. Research should assess for evidence of comorbid anxiety when examining associations between sleep and bipolar disorder. Future research should explore the mechanisms by which comorbid anxiety may contribute to subjective sleep disturbances in bipolar disorder using neurophysiological measures of sleep (i.e., polysomnography).

## Highlights

• Comorbid anxiety is common in people with bipolar disorder.
• We found that subjective sleep quality is reduced in those with comorbid anxiety.
• Comorbid anxiety was not associated with worse sleep as measured using actigraphy.
• This discrepancy could be due to several factors, e.g. sleep state misperception.
• More attention to the effects of anxiety on sleep is needed in this population.

## 1 Introduction

Sleep disturbance forms part of the diagnostic criteria for mood episodes in bipolar disorder - insomnia and hypersomnia (excessive sleep or sleepiness) are symptoms of depression, and “reduced need for sleep” is a symptom of mania (American Psychiatric Association, 2013). However, there is evidence that sleep disturbances in people with bipolar disorder persist during periods of euthymia (Ng et al., 2015). For example, Kanady and colleagues found that sleep disturbances, particularly insomnia, persisted over a 5-year period in individuals with bipolar disorder (Kanady et al., 2015). This is important because sleep disturbances in this population are associated with impaired quality of life and cognitive functioning, in addition to other health implications (e.g., physical inactivity, poorer dietary habits, and substance/alcohol misuse) (Harvey et al., 2009). Furthermore, sleep loss has been implicated as a trigger of mood episodes (Lewis et al., 2017), associated with a poorer course of illness (Eidelman et al., 2010), and is considered a possible risk factor for the onset of bipolar disorder in healthy individuals (Ritter et al., 2011).

Sleep disturbances are also common in people with anxiety disorders, with subjective reports and polysomnographic studies demonstrating a high prevalence of poor sleep quality in panic disorder, post-traumatic stress disorder, and generalised anxiety disorder (Cox and Olatunji, 2016). Sleep problems have also been found to predict the onset of an anxiety disorder (Alvaro et al., 2013) and many anxiety disorders (e.g., generalised anxiety disorder) have sleep disturbances such as insomnia as key diagnostic criteria (American Psychiatric Association, 2013). Likewise, research estimates that 51% of individuals with bipolar disorder experience some form of anxiety disorder comorbidity during their lifetime (Simon et al., 2004). High rates of comorbid anxiety disorder may influence sleep quality in people with bipolar disorder, and may contribute to the high prevalence of sleep disturbances previously found in interepisode periods (Ng et al., 2015). This has important implications both for research aiming to understand the connection between sleep disturbances and bipolar disorder, and for the clinical management of patients. Therefore, it is important to determine risk factors for sleep problems during the inter-episode period that could be a target for interventions aiming to reduce risk of relapse, in addition to improving quality of life.

The present investigation aimed to examine the relationship between sleep disturbances and comorbid anxiety disorder in individuals with bipolar disorder. We assessed bipolar I and bipolar II participants who were currently euthymic and were either experiencing, or not experiencing, a current comorbid anxiety disorder. Due to previously identified discrepancies between objective and subjective measures of sleep (Harvey and Tang, 2012), we utilised a self-reported sleep measure in addition to assessing sleep using actigraphy. We hypothesised that individuals with comorbid anxiety would experience greater sleep disturbance, relative to individuals without a comorbid anxiety diagnosis.

## 2 Methods

### 2.1 Participants

Participants were 101 individuals (n = 65 female) recruited to a Health and Care Research Wales funded research centre, the National Centre for Mental Health (NCMH) research cohort. The centre is a collaboration between Cardiff, Swansea and Bangor Universities, the National Health Service (NHS) and individuals with lived experience of mental health conditions.

Participants were recruited into the NCMH cohort using a range of systematic approaches through primary, secondary, and tertiary health care services including (a) the identification of potential participants by clinical care teams and (b) screening of clinical notes. Participants were also recruited via non-systematic approaches including third-sector organisations and through local/national media outlets. All procedures involving human subjects were approved by Wales Research Ethics Committee 2.

Written informed consent was obtained from all participants. Trained researchers conducted an interview to ascertain participants' history of mental and physical health as well as demographic information and lifestyle. Participants were also asked to complete an additional pack of validated self-report questionnaires in their own time.

### 2.2 Sleep study design

**Fig. 1:**
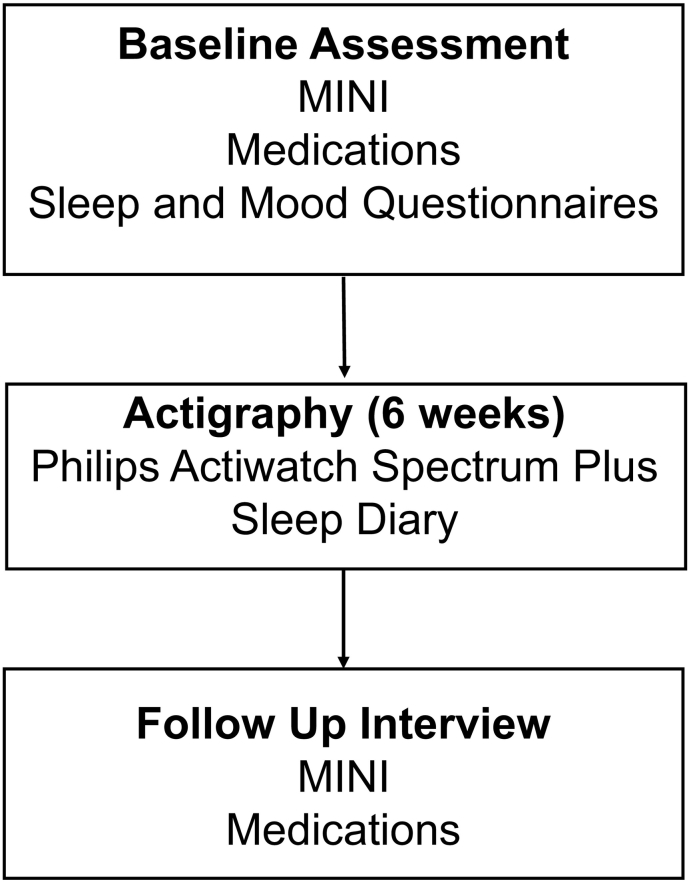
Summary of assessments in NCMH Sleep Study. MINI: Mini International Neuropsychiatric Interview.

Between March 2016 and November 2016, a total of 368 adults currently enrolled in the NCMH cohort who were over the age of 18, had self-reported a diagnosis of bipolar disorder and lived in Wales were invited to participate via an invitation letter. In total, 145 individuals expressed an interest in participating and were screened for eligibility. For the purpose of this study, the presence of bipolar disorder was defined according to DSM-5 diagnostic criteria, based on the information provided at baseline interview (described below) (American Psychiatric Association, 2013). An additional inclusion criterion was self-reported current euthymia, meaning participants were not currently experiencing an episode of depression or high mood. Exclusion criteria included a self-reported diagnosis of rapid cycling or schizoaffective illness, changes in medication within the last two months and travel across three or more time zones within the last two months. A total of 127 participants consented to participate in the NCMH sleep study. Of these, 101 were included in the analysis (additional exclusions outlined in Section 3.1). The protocol for the study is outlined below and summarised in Fig. 1.

#### 2.2.1 Baseline assessment

##### 2.2.1.1 Mini International Neuropsychiatric Interview (MINI)

Eligible participants completed a structured psychiatric interview to confirm a diagnosis of bipolar disorder (bipolar disorder type 1 [BD-I], bipolar disorder type 2 [BD-II], schizoaffective disorder bipolar subtype or bipolar disorder not otherwise specified) using an adapted version of the Mini International Neuropsychiatric Interview (MINI) (Sheehan et al., 1998). The MINI was administered by trained research psychologists (KJSL, HP). Best-estimate lifetime diagnoses were made based on the MINI data by two members of the NCMH team: a research psychologist (HP) and a psychiatrist (IJ) and consensus was reached through discussion where necessary. For inclusion in analyses, participants were required to meet criteria for BD-I or BD-II. The MINI was also used to assess current diagnoses of panic disorder (PD) and generalised anxiety disorder (GAD) according to DSM-5 (American Psychiatric Association, 2013) diagnostic criteria. The presence of anxiety disorder was defined as a diagnosis of GAD and/or PD according to the DSM-5 diagnostic criteria. We focused solely on GAD and PD as these are the most prevalent anxiety disorder comorbidities in individuals with bipolar disorder (Nabavi et al., 2015). Likewise, these diagnoses have previously been associated with sleep disturbances, whereas the association between sleep disturbances and other anxiety disorders (e.g., social anxiety disorder) are less well supported by reliable empirical evidence (Cox and Olatunji, 2016).

##### 2.2.1.2 Medication

During the baseline interview, participants self-reported all current medications (prescribed and “over the counter”), their typical daily dosages and the start date of each drug. Medications that participants reported they were taking were allocated into the following classification groups for analysis: antidepressants, antipsychotics (atypical and typical variants), mood stabilisers (including lithium), as well as anxiolytics (diazepam, temazepam, clonazepam) and sleep medications (zopiclone). These medications have previously been shown to have varying effects on sleep (e.g., some antipsychotics can promote sedation and improve sleep quality, whereas certain antidepressant medications can impair sleep) (Holshoe, 2009; Monti and Monti, 2004).

##### 2.2.1.3 Sleep Quality Questionnaire

Sleep quality was assessed using the Pittsburgh Sleep Quality Index (PSQI) (Buysse et al., 1989), a questionnaire that assesses sleep disturbance (e.g., delayed sleep onset, reduced total sleep time, and poor sleep quality) over a one-month period. Scores range between 0 and 21, with larger scores indicating poorer overall sleep quality (Buysse et al., 1989). The PSQI has been shown to have high sensitivity (89.6%) and specificity (86.5%) for identifying sleep disturbances (Buysse et al., 1989).

##### 2.2.1.4 Mood questionnaires

Current (hypo)manic symptoms were assessed using the Altman Self-rating Mania Scale (AMS), a questionnaire designed to identify (hypo)manic symptoms. It has been shown to have both high sensitivity (85.5%) and specificity (87.3%) when assessing high mood (Altman et al., 1997). Containing five questions, each assessing specific symptoms of mania according to DSM-IV criteria, the questionnaire produces a severity value between 0 and 20, with larger values indicating more severe high mood over the past week. Standardised threshold scores were also calculated, with severity values >5 indicating the presence of high mood (Altman et al., 1997).

Current depressive symptoms were assessed using one of the Beck Depression Inventory-II (BDI-II) (Beck et al., 1996) or the Patient Health Questionnaire-9 (PHQ-9) (Kroenke et al., 2001) as, due to participant feedback about the BDI-II, the latter was introduced as an alternative measure of depressed mood during the study period. The BDI-II is a self-report questionnaire consisting of 21 questions aimed at identifying the presence and severity of a participants' depressive symptoms (Beck et al., 1996) according to DSM criteria. Scores range between 0 and 63, with larger values indicating more severe depressive symptoms. The BDI-II has been shown to have high sensitivity (>80%) and specificity (>70%) when assessing depressive episodes (Wang and Gorenstein, 2013).

The PHQ-9, like the BDI-II, aims to identify the presence and severity of participants' depressive symptoms, and has been shown to have high sensitivity (74%) and specificity (91%) when assessing major depression diagnoses (Arroll et al., 2010). The PHQ-9 contains 9 questions, each targeting individual symptoms of depression according to the DSM-IV. Total scores range between 0 and 27, with larger values indicating more severe depressive symptoms.

The PHQ-9 and BDI-II have previously demonstrated substantial agreement (κ = 0.73, *P* < 0.001) when assessing depression symptoms (Titov et al., 2011) but in order to account for the two measures of depressive symptoms we employed, a standardised score was produced based on validated cut-off thresholds (PHQ-9 ≥ 10 or BDI-II ≥ 11) from each questionnaire (Gilbody et al., 2007; Kroenke et al., 2001; Titov et al., 2011).

#### 2.2.2 Actigraphy

Wrist-based actigraphy is a method for assessing patterns of movement over time. The patterns of movement can be used to infer sleep and wakefulness with good reliability and validity, especially when combined with self-reported sleep diaries (Martin and Hakim, 2011; Tahmasian et al., 2013). For this investigation, we used the Phillips Respironics' Actiwatch Spectrum Plus actigraph. The Actiwatch Spectrum models have been shown to have high sensitivity (96–97%), low to moderate specificity (39–47%) and high accuracy (81%) relative to polysomnography (Jumabhoy et al., 2019; Kahawage et al., 2020). Participants were instructed to wear the Actiwatch Spectrum Plus on their non-dominant wrist every day and night for a period of six-weeks whilst also completing a daily sleep diary. Sleep diaries were completed each morning, with participants reporting the times they got into bed the previous night, fell asleep, woke up and got out of bed. Activity parameters were set to a 60-second epoch length. Participants were required to have at least 7 consecutive days of actigraphy data in order to be included in analysis, adhering to current conventions and best practices for actigraphy-based research (Sadeh, 2011). Data were analysed using the Philips Actiware version 6.0.9 software and a wake threshold was set at an activity value of 40. Research psychologists visually inspected each participant's data using sleep diaries and an actigraphy screening protocol. Rest intervals were entered using self-reported bedtimes and rise times from sleep diaries. If bedtimes and rise times were not recorded, the Actiware software automatic determination of rest intervals was used and cross-referenced with previous diary times. We then derived key actigraphy variables using the Actiware software algorithms. These were Total Sleep Time (total time spent asleep whilst in bed, TST), Sleep Onset Latency (time between bedtime and falling asleep, SOL), Sleep Efficiency (percentage of time asleep whilst in bed, SE), and Wake After Sleep Onset (time spent awake between falling asleep and awake time, WASO). All variables were measured in minutes, except for sleep efficiency which was measured as a percentage. Intraclass correlation (ICC) analyses indicated moderate to excellent reliability between raters on actigraphy variables (ICC_(TST)_ = 0.97, 95% CI 0.95–0.98; ICC_(SOL)_ = 0.47, 95% CI 0.26–0.63; ICC_(SE)_ = 0.88, 95% CI 0.83–0.92; ICC_(WASO)_ = 0.97, 95% CI 0.96–0.98).

#### 2.2.3 Follow-up assessment

At the end of the six-week sleep monitoring period, a follow up assessment was conducted using the MINI to determine whether participants had experienced mood episodes, generalised anxiety disorder or panic disorder during the study period according to DSM-5 diagnostic criteria. Any changes to medication since the baseline assessment were also recorded.

### 2.3 Analysis

We conducted regression analyses for both subjective sleep quality and actigraphy assessments using R version 4.0.2 (R Core Team, 2021), adhering to a *P* < 0.05 criterion for statistical significance. Multiple univariate analyses were conducted, each utilising anxiety disorder diagnosis (diagnosis present, or no-diagnosis present) as the predictor variable. Outcome variables included PSQI global score, Total Sleep Time (TST), Sleep Efficiency, Sleep Onset Latency (SOL), and Wake After Sleep Onset (WASO). Univariate analyses aimed to identify relationships between the predictor and outcome variables.

Where univariate analyses revealed significant associations, we conducted sensitivity analyses controlling for potential confounders. Covariates were chosen based on their previous associations with sleep outcomes in individuals with bipolar disorder. For subjective sleep quality, chosen covariates were bipolar disorder subtype (bipolar I or bipolar II) (Steinan et al., 2016), age (Robillard et al., 2013), gender (Saunders et al., 2015), medication use (including antidepressants, anxiolytics and sleeping medication, mood stabilisers, and antipsychotics) (Holshoe, 2009; Monti and Monti, 2004), depressive symptoms (measured using the BDI-II or PHQ-9) (Beck et al., 1996; Kroenke et al., 2001), and high mood symptoms (measured using the AMS) (Altman et al., 1997). The same covariates were controlled for in sensitivity analyses for actigraphy assessments, with the addition of mood episode occurrence during the sleep monitoring period.

## 3 Results

### 3.1 Sample characteristics

Sixteen participants were excluded due to missing data on key outcome and/or predictor variables, including gender, current mood states, and sleep quality scores. A further two participants were excluded due to less than 7 consecutive days of actigraphy data being present, and another participant was excluded due to being an overnight shift worker. This resulted in 101 participants (n = 81 [80.2%] bipolar I, n = 20 [19.8%] bipolar II) who were included in the analysis. Participants were an average age of 50 years (SD = 11.46), with 65 (64.4%) being female. Within our sample, 44 (43.6%) participants received a comorbid diagnosis of an anxiety disorder according to DSM-5 criteria (1), 16 (15.8%) with panic disorder, 10 (9.9%) with generalised anxiety disorder, and 18 (17.8%) with both panic and generalised anxiety disorders. During the study, 98 (97%) participants were using psychotropic medication. These medications included mood stabilisers (n = 65; 64.4%), atypical and/or typical antipsychotic medication (n = 59; 58.4%), antidepressant medication (n = 58; 57.4%), as well as anxiolytics and sleeping medication (n = 41; 40.6%). Participants average scores on AMS, PHQ, and BDI measures at baseline, were 3.23 (SD = 3.62), 7.77 (SD = 8.80), and 6.18 (SD = 10.58), respectively, with 60 (59.4%) participants scoring above the PHQ/BDI threshold for depression, and 18 (17.8%) participants scoring above the AMS threshold score for high mood. From the MINI interview data however a lower number of participants (n = 43; 42.6%) reported experiencing a mood episode during the period of sleep monitoring. See Table 1 for further descriptive statistics in individuals with and without comorbid anxiety disorder, and Table 2 for descriptive statistics on objective and subjective sleep measures.

**Table 1:** Demographic and clinical characteristics of participants with and without anxiety disorder diagnoses.

Variable	Comorbid anxiety (n = 44)	No comorbid-anxiety (n = 57)	Test statistics	*P*
Age (mean ± SD)	47.93 ± 12.10	51.63 ± 10.77	*t* = 1.60	0.11
Gender, n (%)				
Female	30 (29.70)	35 (34.65)	χ^2^ = 0.25	0.62
Male	14 (13.86)	22 (21.78)		
DSM-5 bipolar diagnosis, n (%)				
Bipolar-I	32 (31.68)	49 (48.51)	χ^2^ = 1.97	0.16
Bipolar-II	12 (11.88)	8 (7.92)		
Medication, n (%)				
Medicated	42 (41.58)	56 (55.45)	χ^2^ = 2.00	0.16
Mood stabilisers	24 (23.76)	41 (40.59)	χ^2^ = 4.45	0.03
Antipsychotics	27 (26.73)	32 (31.68)	χ^2^ = 0.42	0.52
Antidepressants	29 (28.71)	29 (28.71)	χ^2^ = 0.00	0.99
Anxiolytics and Z-drugs	20 (19.80)	21 (20.79)	χ^2^ = 0.02	0.88
AMS score (mean ± SD)	2.64 ± 3.00	3.68 ± 4.01	*t* = 1.50	0.14
PHQ score (mean ± SD)	10.39 ± 10.13	5.75 ± 7.06	*t* = 2.59	0.01
BDI score (mean ± SD)	6.93 ± 12.27	5.60 ± 9.14	*t* = 0.60	0.55
Above PHQ/BDI threshold score,^a^ n (%)	32 (31.68)	28 (27.72)	χ^2^ = 0.27	0.61
Experienced a mood episode during sleep monitoring, n (%)	20 (19.80)	23 (22.77)	χ^2^ = 0.21	0.65

DSM-5, Diagnostic and Statistical Manual of Mental Disorders (5th Edition); AMS, Altman Self-rating Mania Scale; PHQ, Patient Health Questionnaire-9; BDI, Beck Depression Inventory-II.

a Cut-off thresholds defined for PHQ and BDI questionnaires, with above-threshold scores indicating clinically significant depressive mood symptoms: PHQ ≥ 10; BDI ≥ 11.

**Table 2:** Subjective and objective sleep measures in individuals with and without anxiety disorder diagnoses (mean ± SD).

Sleep measures	Comorbid anxiety (n = 44)	No comorbid anxiety (n = 57)	Total sample (n = 101)
PSQI Global score	12.42 ± 4.26	8.96 ± 4.45	10.49 ± 4.68
TST (min)	449.57 ± 80.62	443.58 ± 93.03	446.19 ± 87.47
Sleep efficiency (%)	82.10 ± 7.99	83.46 ± 5.74	82.87 ± 6.81
SOL (min)	20.84 ± 18.96	15.84 ± 10.68	18.02 ± 14.99
WASO (min)	61.85 ± 29.61	56.07 ± 26.37	58.59 ± 27.83

PSQI, Pittsburgh Sleep Quality Index; TST, total sleep time; SOL, sleep onset latency; WASO, wake after sleep onset.

### 3.2 Subjective sleep quality

A comorbid anxiety disorder was associated with poorer sleep quality as measured by the PSQI global score (*R*^2^ (99) = 0.14, B = 3.58, 95% CI = 1.85–5.32, *P* < 0.001). When controlling for potential confounders (bipolar disorder subtype, age, gender, medication use, depressive symptoms, and high mood symptoms) in a multivariate model, anxiety disorder remained strongly associated with PSQI global score (*R*^2^ (89) = 0.31, B = 2.63, 95% CI = 0.92–4.34, *P* = 0.003).

### 3.3 Actigraphy

In contrast to the subjective sleep quality data, presence of an anxiety disorder was not significantly associated with any actigraph measures of sleep disturbance, namely: total sleep time (*R*^2^ (99) = −0.008, B = 5.99, 95% CI = −28.99–40.97, *P* = 0.735), sleep efficiency (*R*^2^ (99) = −0.0001, B = −1.36, 95% CI = −4.07–1.35, *P* = 0.323), sleep onset latency (*R*^2^ (99) = 0.02, B = 5.00, 95% CI = −0.91–10.92, *P* = 0.096), and wake after sleep onset (*R*^2^ (99) = 0.001, B = 5.78, 95% CI = −5.30–16.86, *P* = 0.303).

## 4 Discussion

To our knowledge, this is the first study to use both subjective and objective measures of sleep to examine the association between comorbid anxiety disorders and sleep disturbance in bipolar disorder. We found that presence of a comorbid anxiety disorder was associated with greater self-reported sleep disturbance (even when controlling for covariates known to be associated with poor sleep) but was not associated with objective measures of sleep derived from actigraphy.

### 4.1 Subjective sleep disturbance and anxiety

The finding that comorbid anxiety was associated with higher PSQI scores concords with previous work demonstrating people with anxiety disorders are more likely to report subjective sleep disturbances (Cox and Olatunji, 2016). Given the high rates of comorbid anxiety in bipolar disorder (Simon et al., 2004), future research investigating sleep disturbances in people with bipolar disorder should consider the effect of comorbid anxiety on results. However, due to our study being cross-sectional, we are unable to make conclusions regarding the direction of effect between sleep and anxiety. In other populations, anxiety symptoms have been implicated as a risk factor for insomnia, and insomnia has been shown to increase the risk of developing anxiety, suggesting this relationship is bidirectional (Alvaro et al., 2013).

Several mechanisms could explain the association between anxiety and sleep disturbances. One proposed mechanism is physiological hyperarousal (e.g., increased activity of the hypothalamic–pituitary–adrenal axis), which has been implicated in both anxiety and insomnia disorders (de Lecea et al., 2012; Morin et al., 2015). It has been hypothesised that associations between sleep disturbances and psychiatric disorders may arise due to this overlap (Riemann et al., 2010). Another candidate mechanism is that sleep disturbances increase vulnerability for anxiety disorders by impairing emotion processing (Gruber and Cassoff, 2014). More work is needed to examine these potential mechanisms in individuals with bipolar disorder. In addition, genetic correlations have been observed between insomnia and anxiety disorders (Jansen et al., 2019). Future research might therefore explore how genetic risk factors increase liability to anxiety and sleep disturbances within bipolar populations.

### 4.2 Discrepancies between objective and subjective sleep measures

Discrepancies between subjective and objective sleep measures have been reported previously in research on people with anxiety (Cox and Olatunji, 2016) and bipolar disorder (Harvey et al., 2005), in addition to other psychiatric disorders (e.g. schizophrenia; Tahmasian et al., 2013). Some discrepancies in our study are to be expected, given that the PSQI and the actigraphy were measuring different components of sleep. However, there are a number of other factors that could explain this. The first is “sleep misperception”, a common phenomenon in insomnia research in which subjective poor sleep is not detected by objective sleep measures (Harvey and Tang, 2012). Research is ongoing, but it is thought that worry about sleep and hyperarousal during sleep are contributing factors to sleep misperception (Harvey and Tang, 2012). These may be more likely in people with anxiety and could have contributed to the discrepancies we observe in our study.

A second explanation that has arisen from research on sleep misperception is that objective measures of sleep might not detect aspects of sleep that contribute to the perception of inadequate sleep, such as brief awakenings (between 3 and 30 s) (Harvey and Tang, 2012) and the proportion of time spent in deep sleep (Sadeh, 2011). This is a limitation of actigraphy in particular, as it estimates sleep based on movement data and does not measure sleep neurophysiology. Methods such as high-density EEG and EEG spectral analysis are now revealing aspects of sleep neurophysiology associated with sleep misperception and subjective poor sleep (Lecci et al., 2020; Smagula et al., 2021). This should be explored in future research.

We therefore caution against interpreting the discrepancies between subjective and objective sleep measures as evidence that individuals with comorbid anxiety do not experience real sleep deficits or that perception of sleep quality is not an important clinical measure. First, as highlighted above, subjective and objective measures of sleep are measuring different aspects of sleep. Actigraphy measures sleep using behavioural (i.e., movement) data, therefore whilst it is useful for measuring sleep behaviour over extended periods of time, it has been shown that actigraphy does not always detect aspects of sleep neurophysiology (e.g., spectral EEG characteristics) that contribute to the perception of poor sleep (Smagula et al., 2021). Second, a perception of inadequate sleep is a core diagnostic criterion for sleep disorders such as insomnia and hypersomnia (American Psychiatric Association, 2013). Furthermore, sleep misperception has been associated with poor outcomes, leading researchers to advocate for its treatment (Harvey and Tang, 2012).

### 4.3 Clinical implications

Our results highlight the importance of monitoring and treating both sleep and anxiety in bipolar disorder. In people with bipolar disorder, sleep disturbances have been associated with a worse course of illness (Eidelman et al., 2010), and have been shown to have a negative impact on quality of life and numerous indices of physical and mental health (Harvey et al., 2009). Comorbid anxiety disorders are also common in people with bipolar disorder (Simon et al., 2004) and have been associated with reduced functioning, reduced quality of life and a greater likelihood of attempting suicide (Simon et al., 2004), as well as reduced adherence to medication (Perlis et al., 2010). Sleep disturbances and anxiety can be screened and monitored relatively easily by patients and clinicians, especially with recent advances in digital technology and online monitoring tools (Goodday et al., 2020). In terms of available treatments, cognitive behavioural therapy for insomnia has been shown to improve sleep in addition to mood and functioning in a pilot study of people with bipolar disorder (Harvey et al., 2015). Evidence-based treatments for anxiety disorders are available in clinical practice (Penninx et al., 2021), and some studies suggest that pharmacological and psychological treatments for anxiety in people with bipolar disorder can improve mood and anxiety symptoms, but there is a paucity of research in this area (Yatham et al., 2018). Therefore, treating either anxiety or sleep disturbance may lead to clinical benefit but at present there is insufficient evidence to conclude that one would be more beneficial to treat than the other.

We are not able to determine from our study whether anxiety or sleep disturbances are primary. Longitudinal studies are required in order to determine whether sleep or anxiety disorders are more likely to appear first during illness course. However, monitoring and treating both anxiety and sleep disturbances are important given their aforementioned impact on illness course and quality of life.

### 4.4 Strengths and limitations

To our knowledge, this is the first study to examine associations between comorbid anxiety disorders and subjective and objective sleep measures in individuals with bipolar disorder. Our study design included comprehensive baseline and follow-up assessments, including the MINI interview to identify diagnoses of bipolar disorder and anxiety, and information on medication use. We were also able to account for several potential confounding variables in our analyses, all of which had been previously associated with sleep outcomes. A further strength is that we were able to measure sleep using actigraphy for six-weeks. This enabled the retrieval of more reliable sleep data for each participant and in-turn improved our accuracy when identifying sleeping characteristics (Sadeh, 2011). This contrasts with many existing studies which have used actigraphy only over a one-week period with significantly smaller sample sizes of bipolar disorder participants (De Crescenzo et al., 2017).

Our findings should be considered in light of some limitations. First, we focused on a limited number of comorbid psychiatric disorders, and further research examining other diagnoses that are often comorbid with bipolar disorder (e.g., post-traumatic stress disorder, Cerimele et al., 2017) is needed. Second, the majority of participants (80%) in our sample had a diagnosis of bipolar I disorder, thus future research should further examine participants with bipolar II and those from across the bipolar spectrum (Akiskal et al., 2000). Third, despite being a significant methodological benefit in our study, our objective measures of sleep were limited to actigraphy, which cannot measure sleep neurophysiology. Anxiety disorders have previously been associated with perturbed sleep neurophysiology (Baglioni et al., 2016), therefore, future studies may benefit from using polysomnography to examine differences in sleep between those with and without comorbid anxiety. Fourth, medication and other drug use (e.g. alcohol) can influence sleep (Foral et al., 2011; Garcia and Salloum, 2015). We did not find any differences in medication use between participants with and without comorbid anxiety disorders, including anxiolytics. In sensitivity analyses controlling for covariates, there was weak evidence that participants taking anxiolytic/sleep medication had poorer subjective sleep quality (B = 1.61, 95% CI = −0.03-3.25, *P* = 0.05). However, due to the cross-sectional nature of these data, we cannot infer the direction of effect. In addition, we did not have alcohol consumption data on a sufficient number of participants to include this as a covariate in our analysis. Future research would benefit from a more detailed assessment of the influence of alcohol consumption and medication use – as well as the potential consequence of alcohol misuse on medication nonadherence (Bryson et al., 2008) – on sleep quality.

## 5 Conclusion

Our study indicates that comorbid anxiety in individuals with bipolar disorder is associated with reduced subjective sleep quality but not with sleep measured using actigraphy. This discrepancy could indicate sleep-state misperception or aspects of sleep disturbance that are currently undetectable by actigraphy. Future research should assess for evidence of comorbid anxiety when examining associations between sleep and bipolar disorder and explore the mechanisms by which comorbid anxiety may contribute to sleep disturbances. In clinical practice, comorbid anxiety should be assessed in individuals with bipolar disorder and clinicians should be aware that its presence increases risk of sleep disturbance.

## Funding source

This work was supported by a 10.13039/100010269Wellcome Trust Institutional Strategic Support Fund Consolidator Award and 10.13039/100012068Health and Care Research Wales (Welsh Government). This research was funded in whole, or in part, by the Wellcome Trust [220488/Z/20/Z]. For the purpose of open access, the author has applied a CC BY public copyright licence to any Author Accepted Manuscript version arising from this submission.

## Data availability statement

The data that support the findings of this study are available from the corresponding author (KJSL) upon reasonable request.

## CRediT authorship contribution statement

Conception or design of the work (KJSL, IJ, CL, PG). Acquisition of data (KJSL, HAP, CR). Data analysis (DO). Interpretation of data (KJSL, DO, IJ, CL). Study supervision (KJSL, IJ). KJSL and DO led the drafting of the manuscript, but all authors contributed to the drafting and approved the final manuscript for submission.

## Conflict of interest

PG has research funding from Merck, Inc. and provides consulting to Fisher Wallace Laboratories and Eight Sleep. This work is unrelated to the work in this manuscript.
